# Three-Part MRI Morphology of the Human Masseter Muscle: A Part-Dominance Classification

**DOI:** 10.3390/diagnostics16203398

**Published:** 2026-10-19

**Authors:** Adrian Okoń, Ingrid C. Landfald, Michał Podgórski, Roman Frach, George Triantafyllou, Łukasz Olewnik

**Affiliations:** 1 Department of Clinical Anatomy, Mazovian Academy in Płock, Plac Dąbrowskiego 2, 09-402 Płock, Poland; a.okon@mazowiecka.edu.pl (A.O.); i.landfald@mazowiecka.edu.pl (I.C.L.); 2 Department of Radiology and Diagnostic Imaging, Medical University of Lodz, 90-419 Lodz, Poland; michal.podgorski@umed.lodz.pl (M.P.); roman.frach@umed.lodz.pl (R.F.); 3 School of Medicine, Faculty of Health Sciences, National and Kapodistrian University of Athens, 11527 Athens, Greece

**Keywords:** masseter muscle, anatomical variation, magnetic resonance imaging (MRI), three-part morphology, temporomandibular joint, functional anatomy, classification

## Abstract

**Background:** A simple MRI-based framework for describing part-specific dominance patterns in the human masseter muscle is lacking. This study aimed to characterize its MRI-visible parts and develop a preliminary part-dominance classification. **Methods:** This retrospective cross-sectional study analysed 58 clinical MRI examinations, comprising 116 masseter muscles with complete bilateral visualization. The three MRI-visible parts were identified on T2-weighted 3D coronal source images. Thickness and length were measured using a predefined study procedure, and each muscle was assigned a part-dominance type according to which part was thickest. **Results:** Three distinct parts were identified in all 116 muscles. The inferior part was thickest in 81 muscles (69.8%), the coronoid part in 31 (26.7%), and the middle part in 4 (3.4%). The inferior part was also the longest segment. **Conclusions:** In this dataset, the human masseter muscle showed a three-part MRI morphology. The findings provide a basis for a preliminary part-dominance classification for anatomical and radiological description. Functional, biomechanical, and clinical correlations require prospective validation.

## 1. Introduction

The masseter muscle (MM) is one of the principal muscles of mastication and plays a central role in mandibular elevation, jaw closure, and the generation of masticatory force. It extends from the zygomatic arch to the lateral surface of the mandibular ramus and angle, forming a major structural and functional component of the masticatory apparatus. Traditionally, the MM has been described as a two-layered muscle, consisting of superficial and deep parts, with different fibre orientations and mechanical roles during mandibular movement [1,2,3].

Recent anatomical and imaging studies, however, have challenged this simplified two-layer model. Detailed anatomical investigations have demonstrated that the human MM may exhibit a more complex layered organization, including a distinct deep coronoid component located in close relationship to the coronoid process of the mandible [4,5,6,7]. These findings emphasize that the MM should not be regarded as a uniform structure, but rather as a muscle with a more complex internal organization and part-specific morphology.

Recognition of this complex internal morphology is important because variation in muscle thickness, length, fibre orientation, and relative part dominance may influence the direction and magnitude of force transmission across the mandible. Previous morphometric and biomechanical studies have shown that the size and spatial arrangement of the MM are related to craniofacial morphology and jaw mechanics [1,2,8,9,10]. However, despite increasing interest in masseter architecture, there remains no simple MRI-based classification system for describing part-specific dominance patterns of the human MM in vivo.

Magnetic resonance imaging (MRI) provides a non-invasive method for evaluating soft-tissue architecture and distinguishing internal muscle components. Earlier imaging studies demonstrated the usefulness of CT and MRI for assessing masseter morphology and jaw-muscle cross-sectional dimensions [11,12]. Ultrasound has also been widely applied to MM morphometry, particularly for measuring muscle thickness or volume and examining relationships with craniofacial morphology [13,14,15,16]. MRI is particularly suitable for anatomical evaluation of internal muscle architecture because it allows multiplanar visualization of soft tissues and tendinous septa without ionizing radiation.

The present study aimed to describe the three-part MRI morphology of the human MM using the terms coronoid, middle, and inferior parts as operational descriptors for structured imaging-based assessment. In the present study, the terms middle and inferior are used as operational topographical MRI descriptors and are not intended to represent direct equivalents of the classical deep and superficial anatomical parts. We further propose a preliminary Part-Dominance Classification (PDC-3) based on the thickest MRI-visible part of the muscle. This framework is intended as an anatomical and radiological descriptive tool for structured imaging-based description of masseter morphology and should not be interpreted as a diagnostic or functional classification.

## 2. Materials and Methods

### 2.1. Study Design and Ethics

This retrospective cross-sectional study analysed routine clinical MRI examinations with complete bilateral visualization of the MM. All available MRI examinations acquired during 2025 that fulfilled the predefined eligibility criteria were included. A total of 72 MRI examinations were initially screened. Fourteen examinations were excluded because of image artefacts, incomplete coverage of the masseter muscles, insufficient image quality for reliable delineation of muscle boundaries, previous surgery or scarring involving the masseter region, post-traumatic anatomical changes, pathological changes affecting masseter morphology, or MRI protocol differences that precluded comparable morphometric assessment. The remaining 58 examinations fulfilled the eligibility criteria and were included in the final analysis. No a priori sample-size calculation was performed because of the retrospective and exploratory nature of the study; the final sample size was determined by the number of eligible examinations available during the study period. The cohort comprised 58 examinations, yielding 116 sides, with both sides included for each subject. The study was designed as an anatomical MRI-based morphometric analysis. Clinical referral indications were not used for diagnostic grouping, and no functional diagnosis of bruxism or temporomandibular disorder was analysed.

The study complied with the Declaration of Helsinki and was approved by the Bioethics Committee at the Medical University of Łódź (Approval No. RNN39/25/KE). Owing to the retrospective analysis of anonymised clinical MRI examinations, the requirement for written informed consent was waived by the Bioethics Committee at the Medical University of Łódź (Approval No. RNN39/25/KE). All images were anonymised before analysis.

### 2.2. Eligibility Criteria

Inclusion required complete visualization of both MM and diagnostic image quality sufficient to identify internal muscle architecture. Exclusion criteria were previous surgery or scarring involving the masseter region; post-traumatic changes altering local anatomy, including mandibular fractures; pathological processes affecting masseter morphology, including inflammatory, infectious, or neoplastic changes; incomplete coverage of the masseter muscles within the imaging field; image quality insufficient for reliable delineation of muscle boundaries; pronounced motion-related or magnetic-susceptibility artefacts, including those related to orthodontic appliances; and MRI protocols incompatible with comparable morphometric assessment because of differences in imaging sequence, slice thickness, or imaging plane.

### 2.3. MRI Acquisition

All scans were acquired using a Philips Ingenia 3.0 T MRI system with a Philips dStream 32-channel head coil. Measurements were performed using the integrated distance-measurement tools of IntelliSpace Portal (version 12.1.10; Philips Medical Systems Nederland B.V., Best, the Netherlands).

All primary morphometric analyses were performed on the same T2-weighted 3D coronal source-image dataset. This dataset was used for identification of the three MRI-visible masseter parts, thickness and length measurements, and PDC-3 assignment. The acquisition parameters for this sequence were as follows: repetition time (TR), 2500 ms; echo time (TE), 248 ms; field of view (FOV), 250 × 250 mm; acquisition matrix, 252 × 252; isotropic voxel size, 1 × 1 × 1 mm^3^; slice thickness, 1 mm; acceleration technique, CS-SENSE; acquisition time, 4 min 28 s; and flip angle, 90°.

### 2.4. Definition of Masseter Muscle Parts and Measurement Protocol

The MM was assessed on MRI as a three-part structure comprising the coronoid, middle, and inferior parts. For the purposes of the present study, these terms refer to operational MRI-defined parts identified according to their relative topographical position, fibre orientation, visible tendinous septa, and relationships with adjacent osseous landmarks, including the zygomatic arch, mandibular ramus, and coronoid process. The coronoid part was identified as the deepest superior component located in close relationship to the coronoid process of the mandible. The middle part was identified as the intervening component between the coronoid and inferior parts, with its course assessed relative to the zygomatic arch and mandibular ramus. The inferior part was identified as the most inferior and relatively superficial of the three MRI-defined components. In all examinations included in the study, the tendinous septa separating the three MRI-visible parts were sufficiently and continuously visible to permit their delineation. All primary morphometric measurements were performed directly on the acquired T2-weighted 3D coronal source images, without observer-specific alteration of the imaging plane. These terms were used for structured imaging-based description and do not imply histologically or surgically validated muscular compartments.

Thickness and length were measured for each of the three MRI-visible parts using a predefined study measurement procedure. No multiplanar reformatted images were used for the primary morphometric measurements. Thickness was assessed at the location where the relevant MRI-visible part and its boundaries were most clearly visualized, with the measurement line positioned perpendicular to the local long axis of the part. No predefined anatomical level or fixed osseous landmark was used to select the thickness-measurement location. Accordingly, the measurement position was selected separately for each MRI-visible part according to optimal visualization of its boundaries.

**Figure 1:**
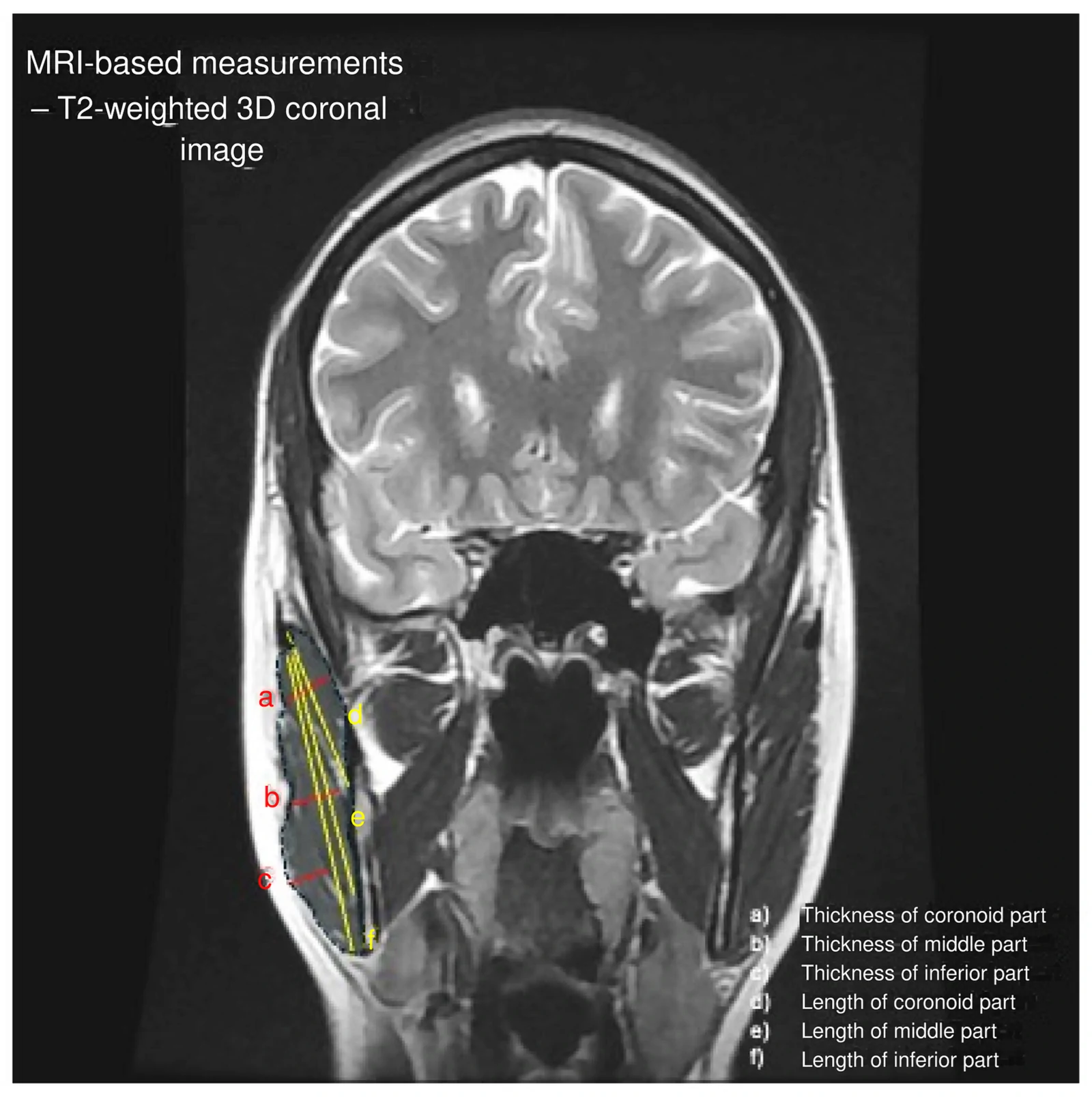
MRI-based measurements of the three masseter muscle parts performed on the T2-weighted 3D coronal dataset used for primary morphometric assessment. a, thickness of the coronoid part; b, thickness of the middle part; c, thickness of the inferior part; d, length of the coronoid part; e, length of the middle part; f, length of the inferior part.

Length was assessed along the visible long axis of each part. All MRI examinations were evaluated independently by three observers (AO, MP, and RF), comprising two radiologists and one anatomist. Each observer independently identified the three MRI-visible parts of the masseter muscle and performed the corresponding thickness and length measurements using the distance-measurement tools integrated into the MRI workstation. The imaging software automatically calculated the corresponding distances after placement of the measurement lines. For each morphometric variable, the final value used in the statistical analysis was calculated as the arithmetic mean of the three independent observer measurements. Each observer assessed each examination once. Because only the mean of the three independent measurements was retained in the analytical dataset, observer-specific values were unavailable for retrospective calculation of interobserver reliability, and no repeated measurements were available for assessment of intraobserver reliability. The measurement approach and identification of the three MRI-visible parts are illustrated in Figure 1.

### 2.5. Part-Dominance Classification

A three-type Part-Dominance Classification (PDC-3) was applied according to the thickest MRI-visible part of the masseter muscle. Type I was defined as inferior-dominant, Type C as coronoid-dominant, and Type M as middle-dominant. The primary PDC-3 type was assigned according to the part with the greatest measured thickness. No minimum absolute or relative difference in thickness between the thickest and second-thickest parts was required for PDC-3 assignment. Consequently, cases with near-equal thickness measurements were classified according to the numerically greater measured thickness. The PDC-3 scheme was developed for the purposes of this study as a preliminary anatomical and radiological framework for describing part-specific dominance patterns of the masseter muscle. In the present classification, the term “dominance” refers exclusively to the MRI-visible part with the greatest measured thickness and does not imply functional, biomechanical, volumetric, or physiological dominance. The classification approach is illustrated in Figure 1.

### 2.6. Statistical Analysis

Statistical analyses were performed using Statistica 13 software (TIBCO Software Inc., Palo Alto, CA, USA). Continuous variables are presented as the median and interquartile range (IQR), as the morphometric variables did not follow a normal distribution. Non-parametric methods were utilized for all inferential statistics. To appropriately account for within-subject dependence resulting from bilateral observations, right and left measurements were averaged for each of the 58 participants, creating a single subject-level observational unit. Differences in thickness and length among the three masseter muscle parts (coronoid, middle, and inferior) were assessed using the Friedman test. Kendall’s W was calculated as a measure of effect size. Following a significant Friedman test, post-hoc pairwise comparisons were conducted using Wilcoxon signed-rank tests. To control the Family-Wise Error Rate (FWER) across the three pairwise comparisons, *p*-values were adjusted using the Bonferroni correction. An adjusted *p*-value of less than 0.05 was considered statistically significant. The distribution of PDC-3 types is presented descriptively as counts and percentages based on sides. Because both sides from the same subject were included, the side was treated as the descriptive unit for the PDC-3 classification, and no formal inferential comparison of type distribution by sex or side was performed.

## 3. Results

The cohort comprised 58 subjects, including 34 females and 24 males. The mean age was 49 ± 17 years in females and 50 ± 18 years in males. Both sides were analysed in each subject, yielding 116 MM.

Three MRI-visible parts of the masseter muscle were identified in all analysed sides: the coronoid part, middle part, and inferior part. Part delineation was based on the position of each part, fibre orientation, and the presence of visible tendinous septa or internal connective tissue planes, as described in the Methods section and illustrated in Figure 1.

The thickness and length of each MRI-visible part are summarized in Table 1. Morphometric analysis at the subject level revealed significant differences among the three MRI-visible parts for both thickness (Friedman test, *p* < 0.001, W = 0.53) and length (Friedman test, *p* < 0.001, W = 1.00). Post-hoc pairwise comparisons using the Wilcoxon signed-rank test with Bonferroni correction demonstrated that the inferior part was significantly thicker than both the middle part (adjusted *p* < 0.001) and the coronoid part (adjusted *p* < 0.001). Furthermore, the coronoid part was significantly thicker than the middle part (adjusted *p* < 0.001). For length, the inferior part was significantly longer than both the middle part (adjusted *p* < 0.001) and the coronoid part (adjusted *p* < 0.001), while the middle part was significantly longer than the coronoid part (adjusted *p* < 0.001).

### Part-Dominance Classification

According to the Part-Dominance Classification (PDC-3), each MM was assigned to one of three types based on the thickest MRI-visible part.

**Figure 2:**
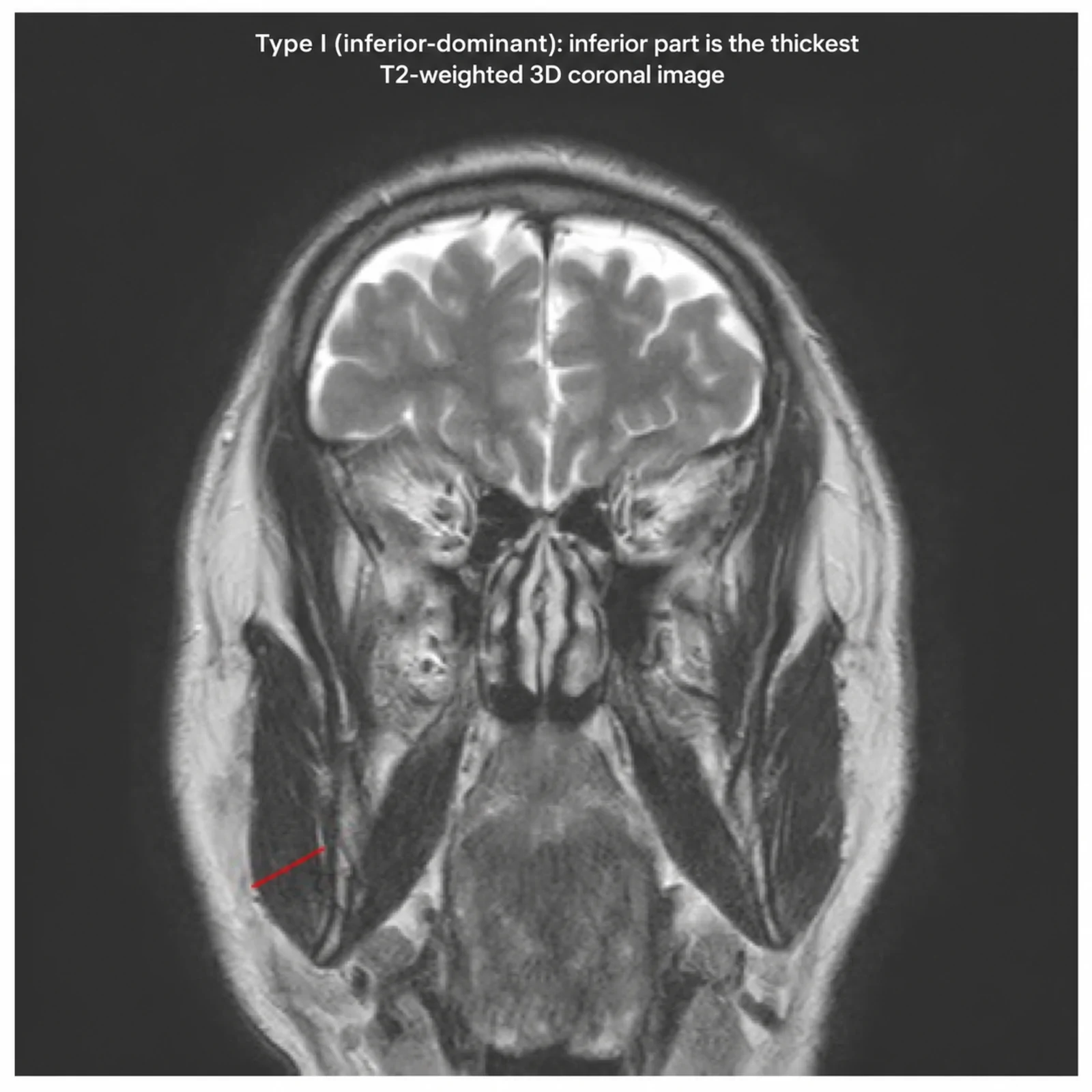
Representative T2-weighted 3D coronal image demonstrating PDC-3 Type I (inferior-dominant), in which the inferior part shows the greatest measured thickness.

Type I, inferior-dominant was identified when the inferior part was the thickest component of the MM. This type was observed in 81/116 sides (69.8%) (Figure 2).

**Figure 3:**
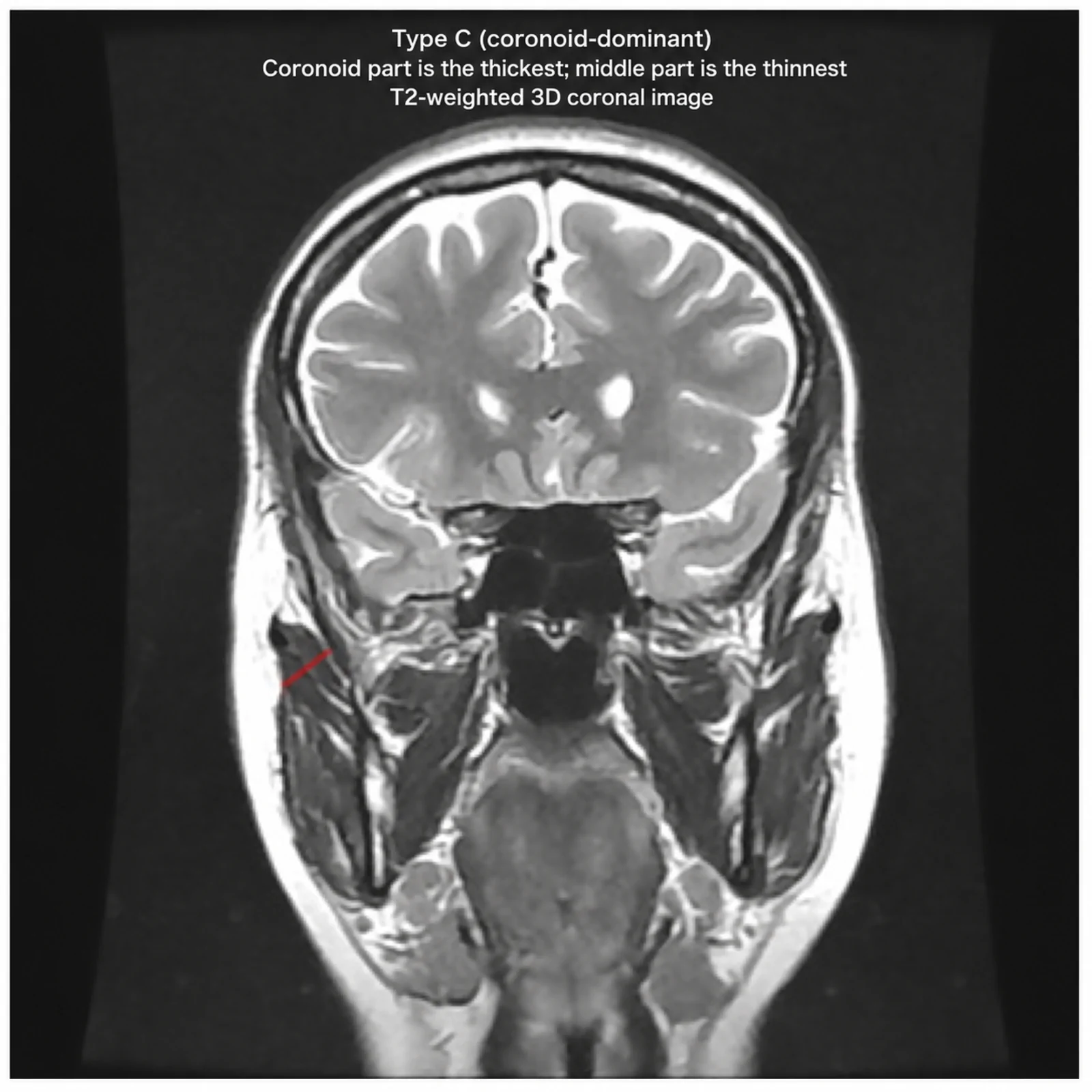
Representative T2-weighted 3D coronal image demonstrating PDC-3 Type C (coronoid-dominant), in which the coronoid part shows the greatest measured thickness.

Type C, coronoid-dominant, was identified when the coronoid part was the thickest component. This type was observed in 31/116 sides (26.7%)—(Figure 3).

**Figure 4:**
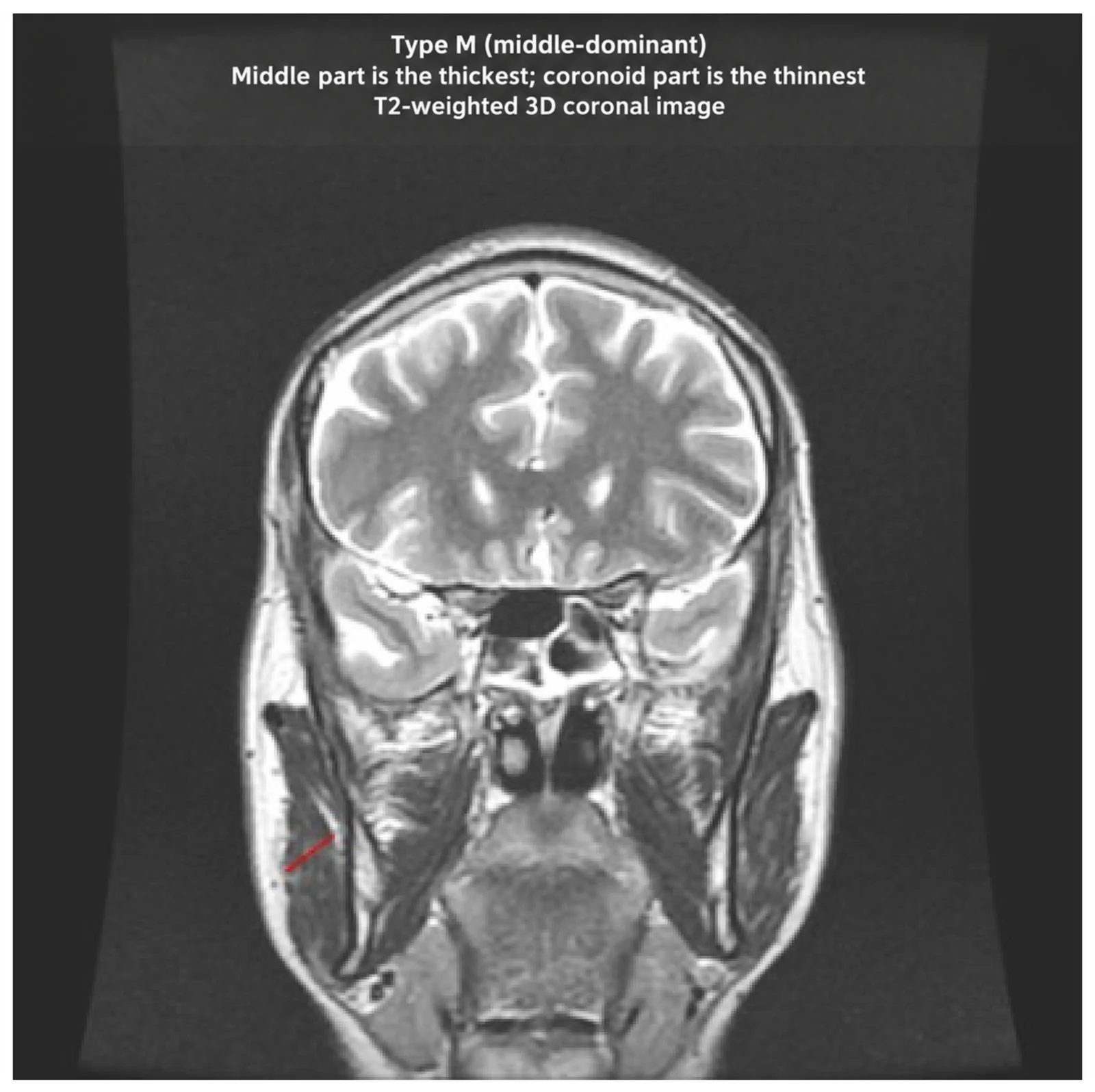
Representative T2-weighted 3D coronal image demonstrating PDC-3 Type M (middle-dominant), in which the middle part shows the greatest measured thickness.

Type M, middle-dominant, was identified when the middle part was the thickest component. This type was observed in 4/116 sides (3.4%)—(Figure 4).

Within the inferior-dominant subset, the middle part was the thinnest component in 51/81 sides (63.0%), whereas the coronoid part was the thinnest component in 30/81 sides (37.0%).

The distribution of PDC-3 types is summarized in Table 2. Because both sides from the same subject were included, the side was treated as the descriptive unit for classification, and no formal inferential comparison of type distribution by sex was performed.

## 4. Discussion

In the present dataset, the human MM was assessed on MRI as a three-part structure comprising the coronoid, middle, and inferior parts. Three MRI-visible parts were identified in all 116 analysed sides using the applied imaging protocol, supporting the view that, within the present dataset, the MM can be described as a structure with a more complex internal organization than a uniform or simply two-layered model. The inferior part was the dominant component in most cases, whereas coronoid dominance was observed in more than one quarter of analysed sides. Middle dominance was rare. These findings provide the basis for a preliminary MRI-based Part-Dominance Classification intended to provide a structured descriptive framework for part-specific MM morphology. The identification of all three MRI-visible parts in every included side should be interpreted in the context of the study design and eligibility criteria. Inclusion required complete bilateral visualization of the masseter muscle and image quality sufficient for assessment of its internal architecture. Accordingly, the 100% identification rate observed in the present dataset should not be interpreted as evidence that an identical three-part configuration is universally identifiable in the general population. Independent anatomical validation and prospective imaging studies are required to establish the generalizability of these observations.

The classical anatomical description of the MM usually distinguishes superficial and deep layers. However, recent anatomical and imaging studies have shown that this simplified model does not fully capture the internal organization of the muscle. Akita and Fukino described the significance of layered structures within the human MM and temporalis muscles, emphasizing that the masticatory muscles demonstrate a more complex architecture than traditionally presented [4]. Similarly, Mezey et al. [5] provided a detailed description of the coronoid part of the human MM using cadaveric dissection, CT, and MRI, further supporting the concept of a distinct deep component associated with the coronoid process [5]. The present MRI-based findings are consistent with this revised anatomical view and indicate that a three-part configuration was identifiable in the present clinical imaging dataset. The PDC-3 framework does not propose a new anatomical subdivision of the masseter muscle. Rather, its distinguishing feature is the use of an MRI-based morphometric approach to classify each analysed side according to which of the three MRI-visible parts shows the greatest measured thickness.

A particularly important observation in this study is the frequency of coronoid-dominant morphology. The coronoid part was the thickest component in 31/116 sides, representing 26.7% of the analysed sample. This suggests that the coronoid component should not be regarded as an occasional or negligible anatomical feature. Instead, its relative development may represent an important source of interindividual variation in MM architecture. Previous reports have described rare coronoid-related anatomical variations in the MM, but the present findings suggest that variation in the relative dominance of the coronoid part may be more common when assessed systematically on MRI [5,17].

From an anatomical perspective, each PDC-3 type may represent a different pattern of internal masseter organization. Type I reflects the most frequent arrangement, in which the inferior part represents the dominant component of the muscle. This pattern is consistent with the traditional emphasis on the superficial masseter as a major contributor to mandibular elevation. Type C is particularly relevant because coronoid dominance indicates that the deep coronoid component may constitute a substantial part of the muscle in a considerable proportion of individuals, supporting recent anatomical descriptions of a distinct coronoid part [4,5]. Type M was rare in the present cohort and should therefore be interpreted cautiously; however, its identification suggests that dominance of the middle component is possible and should be included in a comprehensive imaging-based classification. These types should be regarded as anatomical imaging phenotypes rather than clinical categories.

The proposed PDC-3 framework was designed as a preliminary descriptive approach for capturing part-specific variation in masseter morphology on MRI. The classification does not attempt to define pathological states or functional diagnoses. Rather, it provides an anatomical and radiological language for describing which MRI-visible part of the MM is dominant in a given side. Type I describes inferior dominance, Type C describes coronoid dominance, and Type M describes middle dominance. Such a descriptive system may be useful for future comparative studies, particularly when MM morphology is analysed in relation to craniofacial form, mandibular mechanics, or regional muscle architecture.

The potential biomechanical relevance of this classification should be interpreted cautiously. The size, spatial morphology, and cross-sectional arrangement of the masseter muscle have previously been associated with jaw biomechanics and craniofacial morphology [1,2]. Ultrasonographic studies have also shown that MM thickness varies in relation to facial morphology and can be measured in a standardized way in clinical and research settings [8,15,16]. However, the present study did not include bite force assessment, electromyography, craniofacial measurements, or functional testing. Other studies have examined posture-related differences in masticatory-muscle electromyographic activity [18,19], cervical-muscle morphology in patients with temporomandibular disorders [20], and proposed relationships among dental occlusion, body posture, and temporomandibular disorders [21,22]. These studies provide broader clinical context but do not establish functional correlates of PDC-3. Accordingly, the observed dominance patterns should be considered anatomical imaging phenotypes rather than functional categories. Future studies integrating MRI with functional and morphometric data will be required to determine whether different PDC-3 types correspond to distinct biomechanical profiles.

MRI is particularly valuable for the anatomical assessment of the MM because it provides non-invasive multiplanar visualization of soft-tissue architecture and enables assessment of the spatial relationships between the muscle and adjacent osseous structures. MRI has previously been used to evaluate MM morphology and jaw-muscle dimensions in vivo, while ultrasonography provides an accurate and reproducible alternative for measuring masseter thickness [10,12]. The ability of MRI to visualize deeper soft-tissue structures and internal muscle architecture is particularly relevant to the present study, in which individual MRI-visible parts of the masseter were identified and measured.

The present study has several strengths. First, it provides an in vivo MRI-based assessment of MM morphology using complete bilateral visualization in 58 subjects, yielding 116 analysed muscles. Second, 3.0-T MRI with T2-weighted 3D coronal imaging was used for detailed assessment of the internal architecture of the muscle. Third, the study evaluated the thickness and length of each of the three MRI-visible parts separately rather than treating the masseter as a single morphological unit. Finally, the proposed PDC-3 framework provides a simple descriptive approach that links recent anatomical concepts of three-part masseter organization with in vivo radiological assessment and may facilitate structured and consistent reporting in future anatomical and imaging studies.

The present study also has several limitations. First, it was retrospective and based on a single-centre clinical MRI dataset, which may limit the generalizability of the findings. Second, both sides from each subject were included; therefore, the side was treated as the descriptive unit for classification, and no formal inferential comparison of type distribution by sex or side was performed. Third, the MRI protocol was not originally designed exclusively for masseter morphometry, although complete bilateral visualization and sufficient image quality were required for inclusion. In addition, the MRI-defined parts were not independently validated by cadaveric dissection, anatomical correlation, volumetric segmentation, or another independent reference method. Fourth, the study did not include functional assessment, bite force measurements, electromyography, craniofacial morphometry, or direct clinical correlation. Consequently, no functional or clinical significance of the observed PDC-3 patterns can be inferred from the present data. Although all examinations were assessed independently by three observers and the final morphometric values represented the mean of their measurements, observer-specific measurements were not available in the final analytical dataset. Consequently, formal interobserver reliability could not be calculated retrospectively. In addition, because each examination was assessed only once by each observer, intraobserver reliability could not be evaluated. These limitations preclude conclusions regarding the reproducibility of the proposed classification in the present study. Because the thickness-measurement location was selected according to optimal visualization rather than a predefined anatomical level or fixed osseous landmark, some observer discretion was inherent in the measurement protocol. This should be considered a methodological limitation, particularly because formal interobserver and intraobserver reliability could not be assessed. Because PDC-3 assignment was based on the MRI-visible part showing the greatest measured thickness irrespective of the magnitude of the difference from the second-thickest part, cases with near-equal measurements may have been sensitive to small differences in measured thickness. This represents an additional limitation of the present preliminary classification and should be specifically addressed in prospective validation studies. Accordingly, the present study does not establish a measurement threshold separating unequivocal from borderline part-dominance patterns. Finally, the PDC-3 system should be considered preliminary and requires validation in larger, independent datasets, ideally with interobserver reliability assessment and prospective imaging protocols optimized for masticatory muscle evaluation.

The primary goal of the present study was to characterize the three-part MRI morphology of the human masseter muscle and to propose a preliminary imaging-based framework for describing part-specific dominance patterns. Accordingly, the PDC-3 classification was intended as an anatomical and radiological descriptive system rather than as a diagnostic or functional tool. The present findings provide an initial framework for structured description of masseter morphology in vivo, while the functional, biomechanical, and clinical significance of the identified phenotypes remains to be established in future studies combining imaging with functional and craniofacial assessment.

## 5. Conclusions

This study demonstrates that three MRI-visible parts of the human masseter muscle—the coronoid, middle, and inferior parts—could be identified in the investigated dataset using the applied imaging protocol. The inferior part was the dominant component in most analysed sides, whereas coronoid dominance was observed in more than one quarter of cases and middle dominance was rare. Based on these findings, the proposed Part-Dominance Classification provides a preliminary anatomical and radiological framework for structured description of part-specific masseter morphology in vivo. The PDC-3 system should be interpreted as a descriptive imaging-based classification rather than a diagnostic or functional tool. Independent validation using prospectively defined measurement landmarks and formal interobserver and intraobserver reliability assessment is required before the reproducibility and broader applicability of the PDC-3 framework can be established. Further studies combining MRI with functional, biomechanical, and craniofacial morphometric assessment are required to determine the clinical significance of these morphological patterns.

## Figures and Tables

**Table 1:** Thickness and length of the three MRI-visible parts of the masseter muscle.

Parameter	Coronoid Part	Middle Part	Inferior Part	*p*-Value
Thickness, mm	8.28 (2.33)	6.98 (1.98)	9.63 (1.89)	<0.001
Length, mm	36.88 (6.39)	47.78 (7.13)	65.98 (8.20)	<0.001

Values are presented as median (IQR). Differences between the three parts were assessed using Friedman analysis of variance.

**Table 2:** Part-Dominance Classification (PDC-3) of the MM: counts and percentages.

Subset	Type I Inferior-Dominant	Type C Coronoid-Dominant	Type M Middle-Dominant
Overall (*n* = 116 sides)	81 (69.8%)	31 (26.7%)	4 (3.4%)

Percentages are based on sides. PDC-3, three-type Part-Dominance Classification.

## Data Availability

The data presented in this study are available on request from the corresponding author. The data are not publicly available due to privacy and ethical restrictions associated with clinical imaging data.
